# Research on prescribing cascades: a scoping review

**DOI:** 10.3389/fphar.2023.1147921

**Published:** 2023-07-03

**Authors:** Zhe Chen, Zheng Liu, Linan Zeng, Liang Huang, Lingli Zhang

**Affiliations:** ^1^ Department of Pharmacy, West China Second University Hospital, Sichuan University, Chengdu, China; ^2^ Evidence-Based Pharmacy Center, West China Second University Hospital, Sichuan University, Chengdu, China; ^3^ NMPA Key Laboratory for Technical Research on Drug Products *in Vitro* and *in Vivo* Correlation, Chengdu, China; ^4^ Key Laboratory of Birth Defects and Related Diseases of Women and Children, Sichuan University, Ministry of Education, Chengdu, China; ^5^ West China School of Pharmacy, Sichuan University, Chengdu, China; ^6^ West China School of Medicine, Sichuan University, Chengdu, China; ^7^ Chinese Evidence-Based Medicine Center, West China Hospital, Sichuan University, Chengdu, China

**Keywords:** current status, medication safety, polypharmacy, prescribing cascades, scoping review

## Abstract

**Background:** The concept of prescribing cascades has been proposed for more than 20 years, but the research progress and cognitive level varied in different countries. The aim of this study was to systematically evaluate the current status of relevant original research on prescribing cascades, and to provide references for further research and continuous improvement in clinical practice.

**Methods:** We searched three English databases and four Chinese databases from inception until January 2022. Relevant studies about prescribing cascades meeting the eligibility criteria were extracted independently by two reviewers, and a descriptive analysis was conducted to compare the methods and outcomes of the included studies.

**Results:** A total of 32 studies involving 7,075,200 patients in 11 countries were included, including 13 cross-sectional studies, 11 case reports, 7 cohort studies, and 1 case-control study. The target population was mainly elderly people (24 studies). The purpose of the included studies could be divided into three categories: prevention (4 studies), identification (17 studies), and resolution (11 studies) of prescribing cascades. 49 prescribing cascade routes were identified and mainly attributed to the cardiovascular system, most primary diseases of which were dementia, the initial medications of prescribing cascades were mainly calcium channel blockers, and two to six drugs were involved in the prescribing cascade routes.

**Conclusion:** Prescribing cascades have attracted more attention internationally and current studies have mainly focused on the elderly and their cardiovascular diseases and nervous diseases, but still not yet formed integral research in other special populations of drug use, such as children and pregnant women. It is necessary to further conduct in-depth studies with a broader range, and to establish a series of effective measures to decrease the incidence of prescribing cascades in the high-risk group of drug use.

## Introduction

A prescribing cascade occurs when a new medicine is prescribed to address an adverse drug reaction associated with another medicine, which is correctly recognized as being caused by the offending drug or misinterpreted as a new medical condition requiring treatment. Such a new prescription may increase the possibility of subsequent prescribing cascades (Kalisch et al., 2011; McCarthy et al., 2019). The unrecognized adverse events will cause harmful cascade effects and serious impacts on patient’s health, while the unnecessary risks and costs of additional treatment for patients will greatly increase with the expansion of prescribing cascades. Many commonly used medicines can lead to prescribing cascades, such as non-steroidal anti-inflammatory drugs (NSAIDs), antihypertensive drugs, central nervous system drugs, antibiotics, and anticancer drugs. Approximately 2.9%–8.7% of hospital admissions are associated with adverse drug reactions (Kongkaew et al., 2008; Smyth et al., 2012; Oscanoa et al., 2017), which impose a huge burden on both the medical system and the physical and mental health of patients (Qing-ping et al., 2014; Seo et al., 2023). Currently, there is no recognized method for determining whether a symptom is caused by a medication. However, a clinician can reduce unnecessary medicines, examinations, and injuries through timely identifying prescribing cascades (Piggott et al., 2020).

In some countries, prescribing cascades have been taken seriously and received considerable attention. In 1995, Rochon and Gurwitz first introduced the concept of prescribing cascades in The Lancet (Rochon and Gurwitz, 1995), which was further expanded to British Medical Journal (BMJ) in 1997 (Rochon and Gurwitz, 1997). In 2017, it was refined and revised again in The Lancet (Rochon and Gurwitz, 2017). In 2020, Piggott and his colleagues offered a method to identify prescribing cascades by using clinical flowcharts (Piggott et al., 2020). Conversely, some developing countries seem to have remained unaware of prescribing cascades, for example, Wu et al. (2017) formally introduced the concept of prescribing cascades in China in 2017, and advocated that prescribing cascades should be considered in prescription review.

Against this background, this study aimed to conduct a scoping review of current original research on prescribing cascades and analyze the methodology and results of relevant articles to learn the research advances and development trends worldwide, and provide some references for further study and clinical practice improvement.

## Material and methods

### Search strategy

We searched three English databases including PubMed, Embase, and Cochrane Library, and four Chinese databases including the Chinese Biomedical Literature Database (CBM), VIP Database for Chinese Technical Periodicals (VIP), China National Knowledge Infrastructure (CNKI), and Knowledge service platform of WanFang Data (WangFang) for potentially eligible studies, using (“prescribing cascade*”) OR (prescri* AND cascade*) as the search strategy from database inception to January 2022. Additionally, the reference lists of all included articles were screened for eligible studies. The language was limited to English and Chinese. The detailed search strategies were provided in Supplementary Appendix S1.

### Eligibility criteria and study selection

Studies were selected based on the following inclusion criteria: 1) studies on patients involved in polypharmacy, regardless of gender, age, or race, who were prescribed a new medicine to settle adverse drug reactions, or were receiving treatment that could cause prescribing cascades; 2) evaluation of the process, incidence, or related factors of prescribing cascades as outcomes; 3) study design only in randomized controlled trials, cohort studies, case-control studies, cross-sectional studies, case series studies or case reports. Studies were excluded if they were: 1) inconsistent with the concept of prescribing cascades; 2) duplicate publications; 3) papers that cannot obtain the full text.

Two reviewers independently retrieved and screened the studies according to eligibility criteria using Endnote X9 (Clarivate Analytics, United Kingdom). Disagreements were resolved by discussion with the third reviewer.

### Data extraction and quality assessment

We designed a standardized table and conducted a pre-test with 10% of the included articles to revise the table and formulate instructions for filling it. Data were extracted independently by two reviewers, and the following information was collected: 1) basic information including first author, publication year, country, study design, and study purpose; 2) research methodology, regimens of intervention and control groups, outcomes or observation indicators; 3) sample size, gender, and age of enrolled patients; 4) route and classification of prescribing cascades, including primary disease or symptom, medication, and its adverse effects. Currently, there is lacking the recognized classification standard for prescribing cascade routes, thus we hypothesized the categories of the routes based on the anatomic therapeutic chemistry (ATC) classification of the initial medications of prescribing cascades. Besides, a medication prescribed represents a grade of prescription cascades in the routes. The methodological quality or risk of bias of the included articles was not appraised, which is consistent with the guidelines for scoping reviews (Peters et al., 2015).

### Statistical analysis

Descriptive analysis was performed to investigate the current status of original research on prescribing cascades using Microsoft Excel 2016 (Microsoft Corporation, United States), and the results were displayed in figures and tables.

## Results

### Search results

**FIGURE 1:**
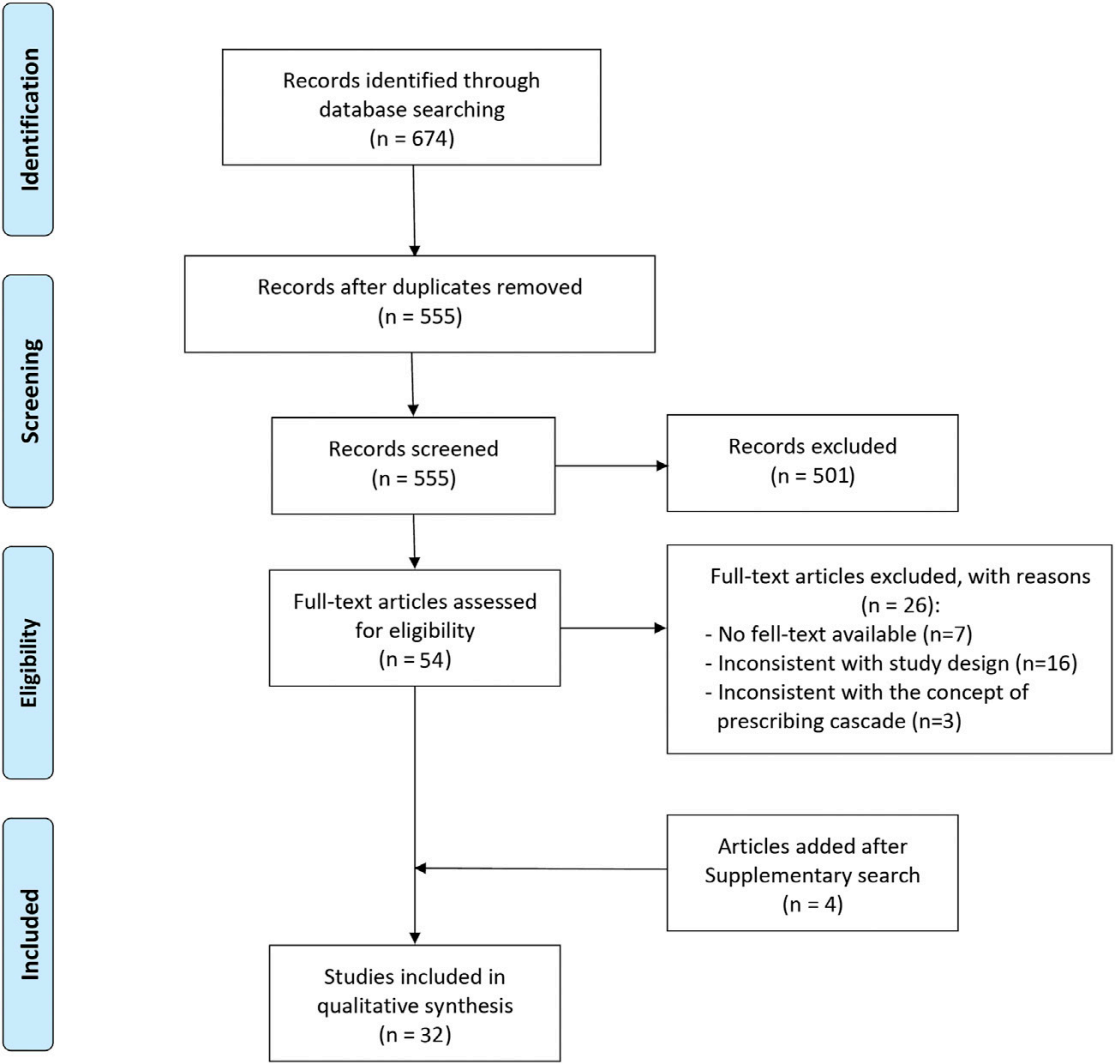
Flow diagram for article selection.

A total of 674 available records were identified from the database search, and 119 duplicate records were removed systematically. After screening the title and abstract, 54 articles were assessed for potential eligibility. 28 articles were selected after reading the full text, and 4 articles were added after reviewing the reference lists above. Overall, 32 studies were eventually included, including 29 English articles and 3 Chinese articles (Figure 1).

### Characteristics of the included studies

32 eligible studies (Gill et al., 2005; Caughey et al., 2010; Vegter and de Jong-van den Berg, 2010; Farrell et al., 2013; Kang et al., 2013; Ribo and Ribo, 2014; Veloso and Cambão, 2015; Hoang et al., 2016; Marras et al., 2016; Nguyen and Spinelli, 2016; Rababa et al., 2016; Ponte et al., 2017; Vouri et al., 2017; Pepa et al., 2018; Vouri et al., 2018; Chi et al., 2019; Huh et al., 2019; Vouri et al., 2019; Bloomstone et al., 2020; Farrell et al., 2020; Hofmann et al., 2020; Savage et al., 2020; Yan et al., 2020; Singh et al., 2021a; Becerra et al., 2021; Singh et al., 2021b; Elli et al., 2021; Masurkar et al., 2021; Morris et al., 2021; Read et al., 2021; Trenaman et al., 2021; Vouri et al., 2022) were identified involving a total of 7,075,200 patients. Among them, 13 were cross-sectional studies (40.63%), 11 were case reports (34.38%), 7 were cohort studies (21.88%), and one was a case-control study (3.13%). All of the included studies were conducted in 2005–2021 and published in the United States (*n* = 12), Canada (*n* = 8), China (*n* = 3), Australia (*n* = 2), Argentina (*n* = 1), Italy (*n* = 1), Republic of Korea (*n* = 1), Netherlands (*n* = 1), Philippines (*n* = 1), Portugal (*n* = 1), and United Kingdom (*n* = 1). The detailed characteristics of the included studies are shown in Supplementary Table S1.

Except for one study (Hoang et al., 2016) that did not report the age and gender of the enrolled patients, of the 31 included studies, 24 studies (77.42%) focused on the elderly, 6 studies (19.35%) focused on adults (≥18 years old), and one study (3.23%) reported on an adolescent aged 14 years. The gender distribution is predominantly female (male/female = 3,072,048/3,816,057) (Supplementary Table S1).

### Purpose and methodology of the included studies

The purpose of the 32 included studies can be divided into three categories: prevention (4/32, 12.50%), identification (17/32, 53.13%), and resolution (11/32, 34.38%) of prescribing cascades (Supplementary Table S1).

Four studies (Ponte et al., 2017; Bloomstone et al., 2020; Farrell et al., 2020; Morris et al., 2021) were relevant to the prevention of prescribing cascades. Ponte et al. (2017) developed a score table and algorithm to detect potential prescribing cascades. Bloomstone et al. (2020) used focus group interviews to evaluate the effectiveness of educational materials on prescribing cascades. Farrell et al. (2020) used semi-structured interviews to investigate the cognition of the concept of prescribing cascades in clinicians, patients, and their caregivers. Morris et al. (2021) measured the quality of life using the relevant tools among patients that experienced prescribing cascades.

Seventeen studies (Gill et al., 2005; Caughey et al., 2010; Vegter and de Jong-van den Berg, 2010; Hoang et al., 2016; Marras et al., 2016; Rababa et al., 2016; Vouri et al., 2018; Huh et al., 2019; Vouri et al., 2019; Savage et al., 2020; Singh et al., 2021a; Singh et al., 2021b; Elli et al., 2021; Masurkar et al., 2021; Read et al., 2021; Trenaman et al., 2021; Vouri et al., 2022) were relevant to the identification of prescribing cascades. Sixteen studies retrospectively analyzed the patient databases, among which Vouri et al. (2019), Vouri et al. (2022), Caughey et al. (2010), Vegter and de Jong-van den Berg (2010), and Vouri et al. adopted the sequence symmetry analysis (SSA) to detect adverse drug events, while Hoang et al. (2016) especially collected the social network media data to mine prescribing cascade signals, aiming to identify and evaluate characteristics, incidence and relevant factors of prescribing cascades.

Eleven studies (Farrell et al., 2013; Kang et al., 2013; Ribo and Ribo, 2014; Veloso and Cambão, 2015; Nguyen and Spinelli, 2016; Vouri et al., 2017; Pepa et al., 2018; Chi et al., 2019; Hofmann et al., 2020; Yan et al., 2020; Becerra et al., 2021) were relevant to the resolution of prescribing cascades. The researchers described the interventions that can reduce the adverse impact of prescribing cascades by reporting typical cases for increasing vigilance for prescribing cascades in clinical work.

### Characteristics of prescribing cascades routes

#### Category

A total of 49 prescribing cascade routes were discussed in the included studies, and 8 different categories were identified according to the classification principle as defined above, including 19 cardiovascular system therapeutic routes (19/49, 38.78%), 16 nervous system therapeutic routes (16/49, 32.65%), 6 alimentary tract and metabolism therapeutic routes (6/49, 12.24%), 3 anti-infective therapeutic routes (3/49, 6.12%), 2 antineoplastic and immunomodulating agents therapeutic routes (2/49, 4.08%), 1 Genito-urinary system and sex hormones therapeutic route (1/49, 2.04%), 1 musculoskeletal system therapeutic route (1/49, 2.04%), and 1 systemic hormonal preparations therapeutic route (1/49, 2.04%). The specific routes of each study were shown in Table 1.

**TABLE 1:** Lists of the prescribing cascades routes in the included studies.

Author	Category	Primary disease or symptom	Prescribing cascades routes
Ponte et al. (2017)	Alimentary tract and metabolism	Nausea	Metoclopramide→sustained dystonia→lorazepam → excessive sedation→trihexyphenidyl
	Antineoplastic and immunomodulating agents	Acute myeloid leukemia	Chemotherapy→febrile neutropenia→imipenem→chills and high fever→NSAIDs
	Alimentary tract and metabolism	Nausea	Metoclopramide→generalized tonicclonic seizure→antiepileptic agents
	Antiinfectives for systemic use	Abdominal symptoms	Imipenem→generalized tonicclonic seizure→lorazepam and phenytoin→ meropenem→generalized tonicclonic seizure
	Antiinfectives for systemic use	Urinary tract infection	Imipenem→central nervous system symptoms exacerbation→valproic acid→hyperammonemia→levetiracetam
	Cardiovascular system	Atrial fibrillation	Amiodarone→hypothyroidism→levothyroxine
	Alimentary tract and metabolism	Epigastric pain and nausea	Metoclopramide→diarrhea→loperamide
	Antineoplastic and immunomodulating agents	Breast cancer	Lapatinib→hypertriglyceridemia→Bate lipid-lowering agents→nausea and vomit →metoclopramide
Bloomstone et al. (2020)	Cardiovascular system	Hypertension	CCB→edema→loop-diuretics
Farrell et al. (2020)	Cardiovascular system	Congestive heart failure	Amlodipine→edema→furosemide→urinary frequency→tamsulosin and dutasteride
	Cardiovascular system	Angina	Diltiazem →edema→chlorthalidone →hyperglycemia→glyburide
	Cardiovascular system	NR	Amlodipine→edema→furosemide
	Systemic hormonal preparations, excl. sex hormones and insulins	Spinal surgery	Prednisone→hyperglycemia→metformin, diclofenac, misoprostol →need for ulcer prophylaxis→omeprazole
	Musculoskeletal system	Pain	Naproxen→need for ulcer prophylaxis→pantoprazole, vitamin B12→hypertension→hydrochlorothiazide →increased uric acid→allopurinol, duloxetine, tamsulosin→headaches→topiramate
	Nervous system	NR	Quetiapine, paroxetine→tremor →levodopa, paroxetine→hypertension→lisinopril
	Nervous system	Trigeminal neuralgia	Pregabalin, carbamazepine, codeine→confusion→rivastigmine
	Cardiovascular system	Hypertension	Amlodipine→edema→furosemide→urinary frequency
Morris et al. (2021)	Cardiovascular system	Hypertension	CCB→edema→loop-diuretics→urinary frequency and incontinence, dehydration, falls, acute kidney injury, etc.
Read et al. (2021)	Nervous system	Low back pain	Gabapentin→edema→diuretics
Masurkar et al. (2021)	Nervous system	Dementia	AChEI→overactive bladder →antimuscarinic agents
Trenaman et al. (2021)	Nervous system	Dementia	AChEI→overactive bladder →antimuscarinic agents
	Alimentary tract and metabolism	Dementia	Metoclopramide→parkinsonism→antiparkinsonian agents
	Cardiovascular system	Dementia	CCB→edema→diuretics
Savage et al. (2020)	Cardiovascular system	Hypertension	CCB→edema→loop-diuretics
Gill et al. (2005)	Nervous system	Dementia	AChEI→urinary incontinence→anticholinergic agents
Marras et al. (2016)	Nervous system	Depressive disorder	Lithium→tremor→antiparkinsonian agents
Vouri et al. (2019)	Cardiovascular system	NR	CCB→edema→loop-diuretics
Huh et al. (2019)	Nervous system	NR	Metoclopramide/levosulpiride →parkinsonism→levodopa
Elli et al. (2021)	Nervous system	Parkinson disease, cerebrovascular disease, hemiplegia	Antidepressant, benzodiazepine, anti-Parkinson dopaminergic agents→constipation→laxative agents
Singh et al. (2021a)	Cardiovascular system	Alzheimer’s disease and related dementia	CCB→edema→diuretics
Singh et al. (2021b)	Alimentary tract and metabolism	Alzheimer’s disease and related dementia	Antipsychotic/metoclopramide→parkinsonism→antiparkinsonian agents
Rababa et al. (2016)	Alimentary tract and metabolism	NR	Polypharmacy→upset stomach→PPIs/H2 receptor blockers
Vouri et al. (2018)	Cardiovascular system	NR	CCB→edema→loop-diuretics
Caughey et al. (2010)	Nervous system	NR	Thiazolidinedione/cardiac therapy/vasodilator for cardiac/anti-hypertensive-vasodilators/diuretic/peripheral vasodilator/β-blocker/CCB/ACEI/ARB/statin/α-adrenoreceptor antagonist/urinary antispasmodics/NSAIDs/muscle relaxant/allopurinol/probenecid/opioids/anti-epileptic medicine/dopaminergic agents/dopamine antagonist/anti-psychotic/anxiolytic/sedatives/anti-depressants/anti-cholinesterase/anti-histamine systemic→dizziness →prochlorperazine→hip fracture
Vegter and de Jong-van den Berg (2010)	Cardiovascular system	NR	ACEI→persistent dry cough→antitussive agents
Vouri et al. (2022)	Cardiovascular system	NR	CCB→edema→loop-diuretics
Hoang et al. (2016)	Cardiovascular system	NR	NSAIDs→hypertension→antihypertensives→tinnitus, gastritis, urticaria, tachycardia, vomiting, rash, numbness, insomnia, psoriasis, chest pain, gastric reflux, renal failure, vertigo, tremor, nausea, arthralgia, edema, cough, weight gain, difficulty breathing, constipation, pruritus, alopecia, tingling, drowsiness, impotence, panic attacks, stomach disorder, cramps, liver failure, seizures, dizziness, mental depression, impaired memory
	Cardiovascular system	NR	ACEI→persistent dry cough→antitussive agents→seizures, numbness, tingling, pruritus, mental depression, cramps, weight loss, nausea, hypersensitivity, vomiting, stomach disorder, tremor, urticaria, dizziness, gastric reflux, rash, insomnia, edema, hyperglycemia, impaired memory, anxiety, vertigo, asthma, tinnitus, constipation, impotence, chest pain, tachycardia, panic attacks
Becerra et al. (2021)	Nervous system	Restless leg syndrome	Ropinirole→orthostatic hypotension and fall→midodrine
Pepa et al. (2018)	Nervous system	Worsening depression, anxiety, and frequent falls	Risperidone→cognitive decline→memantine→odd affect→dextromethorphan-quinidine→worsened cognition and psychotic symptoms→risperidone dose adjustment →orthostatic hypotension→ fludrocortisone →hypokalemia→potassium chloride
Hofmann et al. (2020)	Nervous system	Bipolar affective disorder, ankle edema	Lithium, furosemide→lithium toxicity→semi sodium valproate→confusion, secondary valproic acid toxicity→benzodiazepines→fall
Nguyen Spinelli (2016)	Cardiovascular system	Hypertension	Amlodipine→edema→furosemide, spironolactone→urinary incontinence→fesoterodine→dry mouth→anetholtrithion→fall
Chi et al. (2019)	Nervous system	Urinary incontinence	Tolterodine→worsening Alzheimer’s disease→memantine, donepezil→urinary incontinence
Kang et al. (2013)	Cardiovascular system	Hypertension, after coronary angiography and stent placement	Xinbao pills, baoxinning, ningxinbao capsules, shexiang baoxin pills, compound cerebroprotein hydrolysate tablets, xuesaitong dispersible tablets →diarrhea and tremor →compound digestive enzyme capsules, lacidophilin tablets, live combined *bacillus subtilis* and *enterococcus* faecium enteric-coated capsules, trihexyphenidyl
Yan et al. (2020)	Genito-urinary system and sex hormones	Prostatic hyperplasia	Solifenacin→constipation→maren ruanjiaonang, lactulose oral liquid→diarrhea, urinary retention and elevated sera creatinine levels
Vouri et al. (2017)	Nervous system	Dementia	Donepezil→rhinorrhea→diphenhydramine→worsening cognitive function
Veloso and Cambao (2015)	Nervous system	Migraine	Metoclopramide, acetaminophen→dystonia of the upper limbs→biperiden→confused, spatially disoriented and with short-term memory disturbance
Ribo and Ribo (2014)	Antiinfectives for systemic use	Urinary tract infection	Ertapenem→visual hallucinations, agitation, disorientation, sleeplessness→quetiapine, valproic acid, donepezil
Farrell et al. (2013)	Cardiovascular system	Hypertension, Hyperlipidemia, type 2 diabetes with diabetic neuroroutey sleep disorder, osteoarthritis pain	Ramipril, amlodipine, metoprolol, rosuvastatin, metformin, amitriptyline, acetylsalicylic acid→edema and diarrhea→furosemide, loperamide →fall, orthostatic hypotension, heartburn→rabeprazole→ vitamin B12 and ferritin deficiency→vitamin B12, ferrous fumarate

Notes: AChEI, acetylcholinesterase inhibitors; ACEI, angiotensin-converting enzyme inhibitors; ARB, angiotensin receptor Ⅱ blockers; CCB, calcium channel blockers; NR, not reported; NSAIDs, non-steroidal anti-inflammatory drugs; PPI, proton pump inhibitors.

#### Primary disease or symptom

Except for 11 routes (11/49, 22.45%) that did not mention the primary diseases or symptoms of the enrolled patients, the remaining prescribing cascade routes were mainly generated from the treatment for dementia (8/49, 16.33%), followed by hypertension (7/49, 14.29%), nausea (3/49, 6.12%), mental disorder (3/49, 6.12%), urinary tract infection (2/49, 4.08%), pain (2/49, 4.08%) and cancer (2/49, 4.08%), and other diseases or symptoms were reported for once respectively (1/49, 2.04%) (Table 1).

#### Initial medication

**FIGURE 2:**
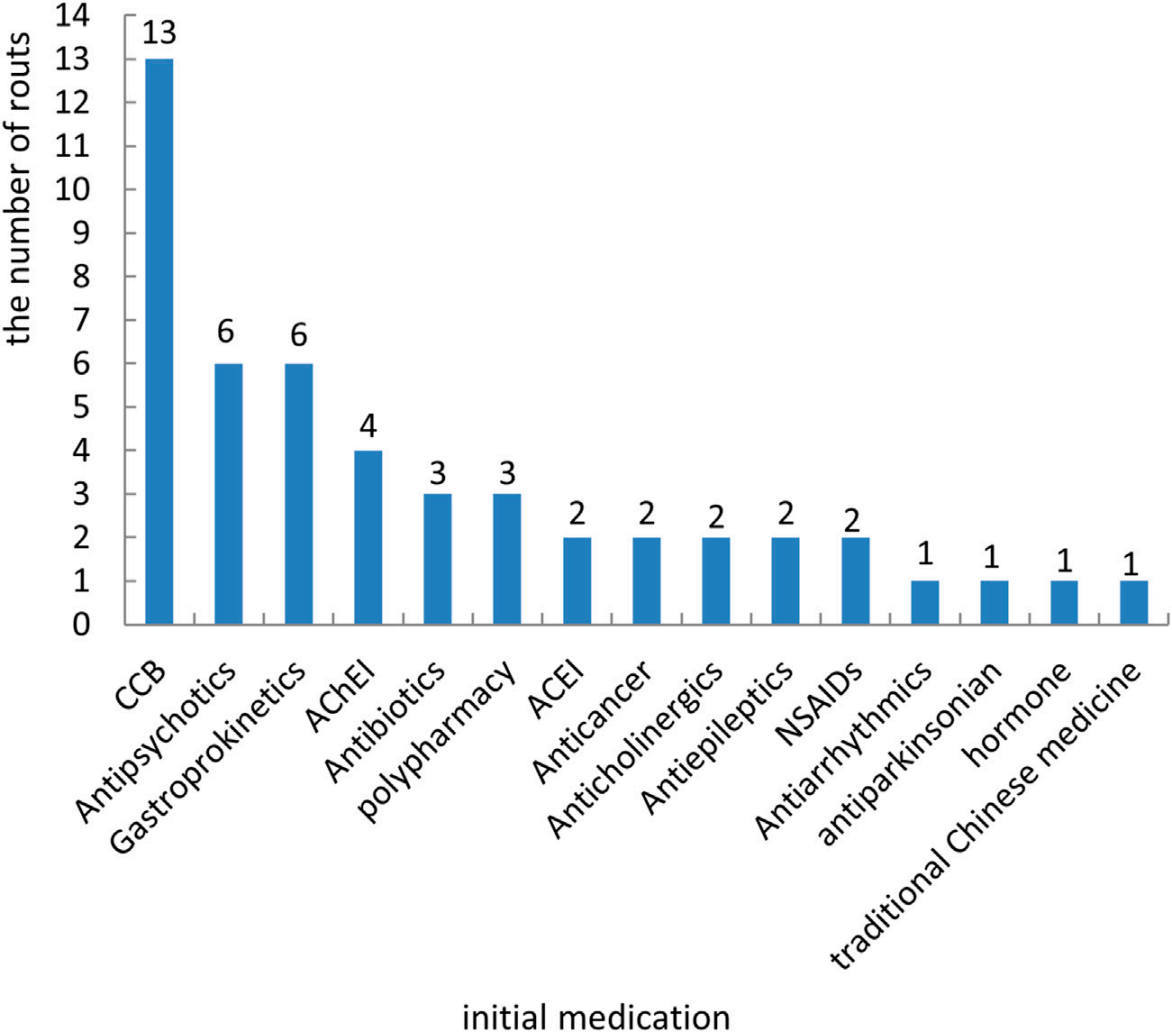
Distribution of the initial medication that caused prescribing cascades. Notes: AChEI, acetylcholinesterase inhibitors; ACEI, angiotensin-converting enzyme inhibitor; CCB, calcium channel blockers; NSAIDs, non-steroidal anti-inflammatory drugs.

Among 49 prescribing cascade routes, the most common initial medication causing prescribing cascades were calcium channel blockers (CCB), with a total of 13 routes (13/49, 26.53%), followed by antipsychotics (6/49, 12.24%), gastroprokinetic (6/49, 12.24%), acetylcholinesterase inhibitors (AChEI) (4/49, 8.16%), antibiotics (3/49, 6.12%), polypharmacy (3/49, 6.12%), and the remaining medications involved 1-2 routes (Figure 2).

#### Grades of medication

The patients enrolled in prescribing cascade studies were prescribed at least 2 and up to 6 kinds of medication, of which a total of 49 prescribing cascade routes all reached the grade second of medication, 14 prescribing cascades routes reached the grade third, 4 prescribing cascades routes reached the grade fourth, and 2 prescribing cascades routes reached the grade fifth and sixth, respectively. Adverse drug reactions leading to new prescriptions included edema, parkinsonism, tremor, diarrhea, constipation, generalized tonic-clonic seizure, overactive bladder, dry cough, etc.

## Discussion

A total of 32 studies were included in this review, and the feasibility of developing continuous research on prescribing cascades was explored by comparing the methods and outcomes of the included studies. The findings showed that the relevant research about prescribing cascades was mainly published in the past 15 years, and the patients enrolled were mostly the elderly. Due to the prescribing cascades occurring in cardiovascular system diseases and nervous system diseases that are more common in the elderly, thus the corresponding medications have attacked more attention than the others.

Elderly patients generally have several chronic diseases, often in the cardiovascular and central nervous systems, rendering medications more complicated than those in adult patients, and decreased physical function accompanied by changes in pharmacokinetics and pharmacodynamics, making the incidence of adverse drug reactions higher than ordinary adults. Thus, the elderly with intricate conditions is undoubtedly considered one of the high-risk groups for prescribing cascades. Previous research indicated that about 8.7% of elderly people were admitted to the hospital due to adverse drug reactions, and NSAIDs and beta-blockers are more frequently associated with these admissions (Oscanoa et al., 2017), which conversely drew less attention in current studies about prescribing cascades.

Only one case report in our review described that the prescribing cascades occurred in a 14-year-old adolescent with migraine (Veloso and Cambão, 2015), which means the prescribing cascades also exist in children with special physiological conditions. Children are in the growth and development stage, whose metabolic function of drugs continuously changes with age as well (Strolin Benedetti et al., 2005). It was reported that the cumulative incidence of adverse drug reactions in hospitalized children is 16.6%, of which about 50% are moderate or severe (Martínez-Mir et al., 1999). Medication in pediatrics still lacks adequate protection (Zhang et al., 2017) and needs should be paid attention to the issue of prescribing cascades in clinical practice, which is currently lacking relevant data for this group. It is recommended that cross-sectional studies are carried out to explore the pediatric incidence of prescribing cascades, supplemented with qualitative studies to analyze the factors contributing to the prescribing cascades, which differ from adults considering the differences in physiological functions and social attributes between the two groups.

Research on prescribing cascades in pregnant women, as one of the high-risk populations of medication, has not been found yet in our review. Such a group also should be very cautious in medication for their dramatic change in pharmacokinetics during pregnancy (Ward and Varner, 2019). da Silva et al. (2019) conducted a cohort study of 1,070 pregnant women and found that the proportion of high-risk pregnant women with adverse drug reactions was 10.7%, which is higher than that of pregnant women with lower gestational age, and the incidence of uncommon adverse drug reactions is less than 0.5%, making those more easily ignored or misinterpreted. There may cause serious consequences and impacts on patients when drug adverse events occur accidentally, thus medical personnel should attach importance to avoiding the prescribing cascades during maternal therapeutics, and the necessity of medication should be considered comprehensively before prescription through improving vigilance.

Besides the target population, our review also found that the current original researches on prescribing cascades have the following deficiencies. Firstly, most clinical studies considered that their results had information bias due to the incomplete diagnostic information of patients enrolled in the retrospective study. It was unable to confirm whether the second-grade medication was exactly prescribed for alleviating symptoms caused by the initial medication, which might be the secondary symptoms of primary disease, other potential diseases, or the same type of adverse reactions originating from another medication. Conditions should be restricted as much rigorous as possible when setting eligible criteria, and more efficient and reliable data collection methods should be adopted. Secondly, prescribing cascades occur in a wide range, but the drug coverage of current studies is not enough to meet the complex requirements of clinical therapeutics and to provide effective evidence for solution developments. The research scope needs to be further expanded to cover more medications for multiple diseases in different conditions. Third, the influence of subjective and objective factors on the incidence and developing process of prescribing cascades was lacking in the simultaneous discussion, such as the ability of clinicians to distinguish between prescribing cascades and adverse drug reactions. The qualitative studies on subjective factors were insufficient and should be also taken into consideration in the future. Fourth, the concept of prescribing cascades was still not yet popularized in some countries, such as China, the Philippines, Portugal, and so on, and the status of the prevalence of prescribing cascades in different regions are needed to be explored and investigated.

It was worth noting that some published studies (Caughey et al., 2010; Vegter and de Jong-van den Berg, 2010; Vouri et al., 2019; Vouri et al., 2022) used the sequence symmetry analysis (SSA) to detect adverse drug events in pharmacological compensation data, which has better sensitivity and specificity than other methods. It can indicate that adverse reactions may be caused by drug A if drug B after drug A is used more frequently than in the past (Lai et al., 2017). SSA often serves as a pharmacovigilance tool to investigate drug safety issues and adverse drug reactions that occur accidentally, which is worth learning in future research, greatly reducing selection bias caused by inadequately rigorous eligibility criteria, such as the inclusion of patients who accept medications due to the secondary symptom of the primary disease. However, its effectiveness may be affected by time shifting, prescription trends, and other factors, resulting in false positive or negative outcomes (Lai et al., 2017), which reminds us that the potential sources of bias should be considered during data analysis.

The identification of prescribing cascades requires clinicians to correctly determine whether the patient’s symptoms were caused by previous medication, and qualitative study can present a more in-depth understanding of early recognition and developing process of prescribing cascades in patients, their caregivers, and medical providers, such as focus group interviews, semi-structured interviews or questionnaire surveys. However, it is still complex in identification due to the processes being simultaneous, non-linear, intersecting, and time-consuming, and healthcare providers have difficulty in assessing the risks and benefits of continuing or deprescribing medications (Farrell et al., 2022). Analyzing driving factors and public perception from multi-perspective may help to provide the foundation for establishing measures or tools to prevent, identify or settle prescribing cascades. The methodology, such as qualitative study and Delphi consensus, can focus more on subjective factors in the real world. But compared to a quantitative study, it is more prone to appear observer or researcher bias, which should be noted to control at the initial design and implementation phase of the study.

The development of supporting tools for the prevention, identification, and resolution of prescribing cascades is necessary for clinical practice. Rational use of drugs is the first step to prevent and identify prescribing cascades, and the prescribing alert systems based on treatment guidelines or drug lists across countries were increasingly developed in recent years, which were one of the effective ways to decrease the incidence of adverse events and prescribing cascades (Gustafsson et al., 2011; Linkens et al., 2023). In addition, it seems feasible that a combination of evidence-based study, qualitative research, and Delphi consensus (Farrell et al., 2022; McCarthy et al., 2022) from multi-perspective including healthcare providers, patients, and their caregivers, can assist to recognize the development of prescribing cascades and establish corresponding intervention measures, and the effectiveness of supporting tools also could be further confirmed through large-scale empirical study.

However, our study also has limitations. We systematically searched 7 databases but limited the search strategies to the definition of prescribing cascades and lacking corresponding MeSH terms that may omit some eligible studies not using such terms, and only included Chinese and English articles, which may introduce language bias. Meanwhile, we excluded conference abstracts, letters, and other non-original studies that were ongoing or discovered cases related to prescribing cascades but had not yet been published as articles. It is recommended that future studies keep up with the latest research on time.

## Conclusion

In conclusion, current research on prescribing cascades has gradually emerged around the world and was mainly concerned with the elderly and their cardiovascular diseases and nervous diseases, but was less attention on other special populations and high-risk groups of drug use, such as children and pregnant women. Further studies combined with multiple methodologies are still warranted to carry out to learn more about the prevalence, characteristics, or related factors of prescribing cascades on patients with different ages and conditions. In addition, cognition and behavior factors that affect the progress of prescribing cascades also should be taken into consideration during the development of tools for the prevention, identification, and resolution of prescribing cascades. Effective measures and interventions are particularly needed to establish for ensuring drug safety in the medication.

## Data Availability

The original contributions presented in the study are included in the article/Supplementary Material, further inquiries can be directed to the corresponding author.
