# Supplementary material for: Research on prescribing cascades: a scoping review
Source: Front Pharmacol. 2023 Jul 3;14:1147921. doi: 10.3389/fphar.2023.1147921 (PMC10350531; doi:10.3389/fphar.2023.1147921)
Supplement: Supplementary file 1 [file Table1.docx]

**Table 1. Characteristics of the included studies in the review**

| **Author** | **Country** | **Study design** | **Methodology** | **Population** | **Sample size** | **Gender(male/female)** | **Age (years)** | **Main findings** |
| --- | --- | --- | --- | --- | --- | --- | --- | --- |
| **1.Prevention** | |  |  |  |  |  |  |  |
| Ponte, 2017 | Argentina | cross-sectional study | prescribing cascade score and algorithm | adults | 8 | 4/4 | 18-67 | - This prescribing cascade score and algorithm are intended to define the problem and to raise awareness among health professionals. - A treatment of ADR is often performed without proper pharmacological knowledge. Since futile poly-medication enhances the chances of developing new diseases, the resulting polypharmacy represents a major health risk, raises senseless health costs, and generates potentially preventable morbidity and mortality. |
| Bloomstone, 2020 | USA | cross-sectional study | focus group interview | adults | 47 | 20/27 | Patients: ≥65 years | - By giving patients and caregivers a basic description of the prescribing cascade concept, the educational materials may help them prepare for a conversation with the provider, who can then tailor the discussion of the possible cascade to the specific needs of the individual patient and caregiver. - Evidence on whether materials can stimulate such conversations awaits testing in a future trial. |
| Farrell, 2020 | Canada | cross-sectional study | semi-structured interview | adults | 14 | Patients: 4/4 | Patients: 70-95 | - Patients and healthcare providers struggled to recognize prescribing cascades and identify when they had occurred; knowledge gaps contributed to this challenge and led to inaction. - Strategies that equip patients and clinicians with resources to recognize prescribing cascades and environmental and social supports that would help with their identification are needed. |
| Morris, 2021 | USA | cross-sectional study | Measurement of quality of life using the Medical Outcomes Study Short Form 12-Item Health Status Survey version 2(SF-12v2) and voluntary self-administered questionnaire (SAQ) | the elderly | 5,458,467 | 2,479,231/2,979,236 | ≥55 years | - This study suggests that a potential dihydropyridine calcium channel blocker (DH CCB)-loop diuretic prescribing cascade may significantly impact physical functioning among older adults. - Given the common use of DH CCBs in the treatment of hypertension and the high frequency of loop diuretic associated adverse events, it is important for clinicians to be aware of this potential prescribing cascade. |
| **2.Identification** | |  |  |  |  |  |  |  |
| Read, 2021 | Canada | cohort study | / | the elderly | 285,481 | 127,408/158,073 | 74(69-80) | - Among older adults, gabapentin use was associated with subsequent diuretic use. The strength of the association increased with increasing gabapentin dosages. - Given the widespread use of gabapentin, the population-based scale of this problem may be substantial. Increased awareness of this prescribing cascade is required to reduce the unnecessary use of diuretics and the exposure of patients to additional adverse drug events. |
| Masurkar, 2021 | USA | cohort study | Andersen Behavioral Model (ABM) | the elderly | 47,059 | 29,472/17,587 | 81.2±5.3 | - Acetylcholinesterase inhibitors (AChEIs) have been associated with an increased risk of antimuscarinic treatment to treat overactive bladder (OAB). About 8% of new users of AChEIs had incident OAB diagnosis or antimuscarinic prescription. - Donepezil use is more likely to lead to antimuscarinic cascade compared with rivastigmine. - There was no differential risk of cascade between galantamine and rivastigmine |
| Trenaman, 2021 | Canada | cohort study | / | the elderly | 28,953 | 10,529/17,946 | 81.1(81.0-81.2) | - The combination of diuretics following CCB was the most common prescribing cascade and bladder anticholinergics following cholinesterase inhibitors the second most common. - Exposure to the inciting medications did not increase risk of exposure to the second treatments. - Combinations of bladder anticholinergics following cholinesterase inhibitors and diuretics following CCBs were more common for women raising concern that women may be at increased risk of these prescribing cascades |
| Savage, 2020 | Canada | cohort study | / | the elderly | 339,019 | 147,334/191,685 | 74.5±6.9 | - Many older adults with hypertension who are newly dispensed a CCB subsequently receive a loop diuretic. - Given how widely CCBs are prescribed, interventions are needed to raise clinicians’ awareness of this common prescribing cascade to reduce the prescribing of potentially unnecessary medications that may cause harm. |
| Gill, 2005 | Canada | cohort study | / | the elderly | 44,884 | 30,099/14,785 | Intervention/ Control (mean): 81.3/82.4 | - The use of cholinesterase inhibitors is associated with an increased risk of receiving an anticholinergic drug to manage urinary incontinence. - Clinicians should consider the possible contributing role of cholinesterase inhibitors in new-onset or worsening urinary incontinence and the potential risk of co-prescribing cholinesterase inhibitors and anticholinergic drugs to patients with dementia. |
| Marras, 2016 | Canada | cohort study | / | the elderly | 288,690 | 86,494/202,196 | 66-87 | - Chronic lithium use is associated with an increased incidence of dopaminergic drug use compared with antidepressants, identifying a prescribing cascade related to lithium use in the elderly. |
| Vouri, 2019 | USA | cohort study | prescription sequence symmetry analysis | adults | 55,818 | 22,902/32,916 | 33,100 aged <65 years;  22,718 aged ≥65 years | - Excessive use of loop diuretics following initiation of DH-CCBs that cannot be completely explained by secular trends or hypertension progression. - The prescribing cascade was more pronounced among those initially prescribed a high dose of DH-CCBs. |
| Huh, 2019 | South Korea | case-control study | / | the elderly | 12,084 | 6,276/5,030 | ≥65 years | - Metoclopramide and levosulpiride were frequently prescribed within 90 days of a prescription for levodopa. |
| Elli, 2021 | Italy | cross-sectional study | / | the elderly | 2,602 | 608/1,994 | 88.4±8.5 | - Medications that can induce constipation, such as antidepressants, anti-Parkinson dopaminergic agents, and benzodiazepines, are often used together with laxatives, and combinations of these drugs further increase the use of laxatives. - Optimizing the prescription of psychotropic drugs could help reduce the "prescribing cascade" with laxatives. The length of stay in NHs is often proportional to laxative use and chronic treatment is very common. |
| Singh, 2021(a) | USA | cross-sectional study | / | the elderly | 121,538 | 45,941/75,597 | 79.5±8.6 | - The proportion of a CCB-diuretic prescribing cascade, widely considered as common and of high importance, was low. This approach can be used to assess the burden of prescribing cascades in large cohorts. |
| Singh, 2021(b) | USA | cross-sectional study | / | the elderly | 121,538 | 45,941/75,597 | 79.5±8.6 | - The proportion of antidopaminergic-antiparkinsonian medication prescribing cascades, widely considered as high-priority, was low in patients with Alzheimer's disease related dementia. |
| Rababa, 2016 | USA | cross-sectional study | / | the elderly | 248 | 78/170 | 88±7.6 | - Evidence supports that PPIs may represent a prescribing cascade in which the PPI is prescribed to counter the negative effects of other drugs and the number of oral products taken. - Because of the high prevalence of long-term PPI use, possible side effects associated with long duration of use, and high financial burden, the need for long-term PPI therapy must be carefully evaluated. |
| Vouri, 2018 | USA | cross-sectional study | / | adults | 47,527 | 23,175/24,352 | ≥18 years | - A potential DH-CCB-associated LEE loop diuretic prescribing cascade was present in approximately 2.2 million patient visits in which DH-CCB was continued. - Older age and an increasing number of concomitant medications were associated with this potential prescribing cascade. |
| Caughey, 2010 | Australia | cross-sectional study | prescription sequence symmetry analysis | the elderly | 34,235 | 16,090/18,145 | 80(76-84) | - Prescribers should consider the possible contributing role of newly initiated medicines with the potential to cause dizziness, and where possible address this through dose reduction or cessation of the medicine, rather than prescribing prochlorperazine. |
| Vegter, 2010 | Netherlands | cross-sectional study | prescription sequence symmetry analysis | the elderly | 1,054 | 408/646 | 65.3±13.9 | - There was a significant and clinically relevant excess of patients receiving antitussives after ACEI initiation. - Cough as a side-effect of ACEI is not recognized as being ACEI-related or is symptomatically treated with antitussive agents instead of ACEI substitution. - The estimated frequency of antitussive treatment of ACEI-induced dry cough is 15%. |
| Vouri, 2022 | USA | cross-sectional study | prescription sequence symmetry analysis | adults | 90 | 30/60 | 60±13.7 | - Over 50% of patients without congestive heart failure were initiated on loop diuretics due to a prescribing cascade. - After removing patients with the confirmed prescribing cascade, the loop diuretic initiation rates before and after DH-CCB initiation were similar, thus confirming the PSSA symmetry assumption. |
| Hoang, 2016 | Australia | cross-sectional study | Data mining and Algorithm framework construction | without limitation | 185,833 | NR | NR | - This study demonstrates the feasibility of generating hypotheses of detrimental prescribing cascades from social media to reduce pharmacists' guesswork. |
| **3.Resolution** | |  |  |  |  |  |  |  |
| Becerra, 2021 | USA | Case report | / | the elderly | 1 | Male | 71 | - This case illustrates two major points. First, this prescription cascade caused by ropinirole, as well as the increase in health care costs attributed to iatrogenic admissions, are major preventable issues faced particularly by the geriatric population. Second, although orthostatic hypotension associated with ropinirole has only been anecdotally reported in patients treated for Parkinson’s disease (higher doses), this side effect should be considered when prescribing ropinirole for other indications, with cautious assessment of risks and benefits. Given the little amount of evidence on ropinirole, further studies will need to be done to assess the frequency at which orthostatic hypotension occurs. |
| Pepa, 2018 | USA | Case report | / | the elderly | 1 | Female | 74 | - This case highlights the importance of maintaining a high degree of suspicion for prescribing cascades in the field of geropsychiatry. |
| Hofmann, 2020 | UK | Case report | / | the elderly | 1 | Male | 69 | - This case is a good example of how one symptom occurs related to one medication, we then prescribe a new drug, leading to a new symptom. This practice may result in increasing polypharmacy and risk of severe adverse effects such as falls. - This case report is intended to improve our level of awareness that new symptoms may be the consequence of the treatments initiated. |
| Nguyen, 2016 | Canada | Case report | / | the elderly | 1 | Female | 71 | - Prescribing cascades attract little attention from clinicians and investigators despite their potential impact on patient’s health and quality of life. |
| Chi, 2016 | China | Case report | / | the elderly | 1 | Female | 87 | - The administration of cholinesterase inhibitors in elderly patients with dementia is associated with a heightened risk of urinary incontinence, while the use of anticholinergic drugs to treat this condition may exacerbate cognitive decline. The prescribing cascade between these two medications is relatively common. |
| Kang, 2013 | China | Case report | / | the elderly | 1 | Male | 65 | - When new symptoms arise during multiple drug therapy in elderly patients, it is imperative to first rule out medication-related factors before considering the addition of another medication for symptomatic relief, thereby preventing the incidence of prescribing cascades. |
| Yan, 2020 | China | Case report | / | the elderly | 1 | Male | 80 | - Throughout the patient's treatment, the entire medication process exhibited typical characteristics of prescribing cascades, with clear causal relationships resulting in unnecessary iatrogenic harm to the patient and prolonging their hospital stay. Timely identification and intervention of prescribing cascades are particularly crucial for ensuring drug safety. |
| Vouri, 2017 | USA | Case report | / | the elderly | 1 | Female | 77 | - This case report serves as a reminder of the necessity of performing a complete medication history to identify potentially harmful medications including over-the-counter (OTC) products, ensure medication causes for new symptoms are ruled out, and intervene on medications used to treat adverse events of other medications (i.e., prescribing cascade). |
| Veloso, 2015 | Portugal | Case report | / | adolescent | 1 | Female | 14 | - The use of acetaminophen is not based on high-quality evidence and better options exist. - Among youngsters, domperidone should be preferred over metoclopramide because it does not cross the blood-brain barrier. - Moderate to severe migraine crises can be managed with triptans in teenagers over 12 years old. - It is important to recognize adverse drug effects. - Harmful consequences of medical interventions do occur. - The school community must be informed about the chronic diseases of the young. |
| Ribo, 2018 | Philippines | Case report | / | the elderly | 1 | Female | 85 | - Ertapenem may exhibit non-seizure neurotoxicity if used beyond recommended dose. |
| Farrell, 2013 | Canada | Case report | / | the elderly | 1 | Female | 81 | - This case highlights how dose reduction and elimination of medications that are no longer needed can significantly improve an older patient’s perception of quality of life in terms of reduced fall risk and increased satisfaction with the medication regimen. |

**Notes:** AChEI: Acetylcholinesterase inhibitors; ACEI: angiotensin-converting enzyme inhibitors; CCB: calcium channel blockers; DH-CCB: dihydropyridines calcium channel blockers; NR: not reported.
